# Extensive Middle Cranial Fossa Arachnoid Cysts and Different Clinical Presentation in Two Patients

**DOI:** 10.4274/jcrpe.1381

**Published:** 2014-09-05

**Authors:** Özge Yüce, Esra Döğer, Nurullah Çelik, Hamdi Cihan Emeksiz, Erkut Baha Bulduk, Mahmut Orhun Çamurdan, Aysun Bideci, Peyami Cinaz

**Affiliations:** 1 Gazi Univesity Faculty of Medicine, Department of Pediatric Endocrinology, Ankara, Turkey; 2 Gazi Univesity Faculty of Medicine, Department of Pediatric Neurosurgery, Ankara, Turkey

**Keywords:** Extensive arachnoid cyst, Growth hormone deficiency, puberty precocious

## Abstract

Arachnoid cysts (ACs), particularly suprasellar cysts, cause a wide spectrum of endocrine disorders. Herein, we report two patients diagnosed with an extensive AC in the middle cranial fossa while being investigated for etiologies of precocious puberty and short stature. One of them required surgery due to his pubertal disorders associated with compression effects of cyst. After surgery, his puberty progression was regressed within one year. On the other hand, surgery was not planned for the second patient considering of his cranial imaging findings and the extremely low incidence of growth hormone (GH) deficiency caused by middle fossa AC (MFAC). We started treatment with recombinant human GH and no complication was found during treatment follow-up. Endocrine disorders associated with MFACs are extremely rare. By presenting with these two cases, we aimed to remain our fellow physcians that ACs can be possibly cause of endocrine disorders. Clinicians should be careful evaluating endocrine disorders because real cause may not be cyst itself but masked by it.

## INTRODUCTION

Arachnoid cysts (ACs) are benign and usually originate from congenital developmental anomalies of the arachnoid membrane. They may also arise after an infection, trauma, or hemorrhage (1). The prevalence is reported to be about 1.7%-2.6% in children (2,3).

ACs make up approximately 1% of intracranial space-occupying lesions and more than 50% of them occur in the middle cranial fossa (2). Many ACs are identified incidentally in children examined for head injury or macrocephaly (4,5). Neurological and visual field abnormalities and diverse endocrine disorders such as central precocious puberty and growth hormone (GH) deficiency have been reported to be associated with ACs, depending particularly on the localization and size of the cyst (6,7,8).

Herein, we present two patients with different endocrine disorders associated with an extensive AC in the middle cranial fossa.

## CASE REPORTS

**Patient 1 **

**Figure 1:**
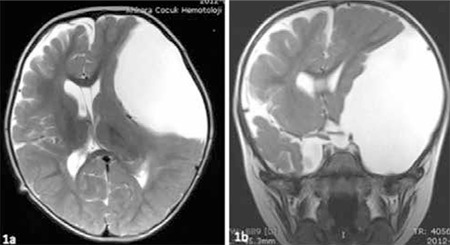
a,b) Axial/ Coronal MRI scan of the patient I showing a large middle cranial fossa arachnoid cyst, its stalk is deviated to the right side with midline shift (T2 weighted image)

An 18-month-old boy presented to the endocrinology clinic with rapid progression of growth and increased head circumference almost since birth. He had been delivered at 38 weeks’ gestation by caesarean section due to breech presentation. Birth weight has been 3000 g and length 50 cm. He was the first child of non-consanguineous parents and his family history was unremarkable. On physical examination, the height was 90 cm [+2.4 standard deviation (SD)] and the weight was 15.5 kg (+2.6 SD). His head circumference was 54 cm (+2.6 SD). The volume of each testis was 4 mL. The psychomotor development was normal. The basal luteinizing hormone (LH) level was high (1.2 mIU/mL). After administration of LH releasing hormone stimulating test, serum LH and follicle-stimulating hormone values rose to peak levels of 7.2 mIU/mL and 2.6 mIU/mL, respectively. Bone age as determined according to Greulich and Pyle method was 3 years. Brain magnetic resonance imaging (MRI) was performed to rule out intracranial lesions. The finding of MRI revealed an unilocular large cyst extending toward the vertex in the left middle cranial fossa (Figure 1a and 1b). The pituitary stalk was deviated to the right due to the compressive effect. A cysto-peritoneal shunt was implemented by neurosurgeons. Postoperatively, a hormonal assay during three months showed decreased LH (0.05 mIU/mL). Pubertal development regressed within 1 year without medical treatment.

**Patient 2 **

A male patient aged 11 years and 6 months was referred to the endocrinology department because of short stature. He was born at 40 weeks’ gestation by normal vaginal delivery. His birth weight and length have been both normal. His growth throughout infancy and childhood was subnormal among peers. He was the second child of non-consanguineous parents and two other siblings were healthy. His family history is unremarkable. He had suffered a scalp laceration and cranial bone fracture after a traffic accident when he was 7 years old. At that time, an AC in the middle cranial fossa had been incidentally detected. On physical examination, his height was 117 cm (-4.3 SD) and the weight was 20 kg (+2 SD). His bone age as determined according to Greulich and Pyle method was 10 years and 6 months. Targeted and predicted height was 172.7±5 cm and 148.2 cm, respectively. Pubertal stage was Tanner stage 1. Laboratory findings, apart from a low level of insulin-like growth factor (IGF)-1 (11 ng/mL; <-3 SD), were almost normal. He had low growth velocity (1 cm/year). We then performed a GH stimulation test. The peak GH level was 6.6 ng/mL with L-Dopa and 5.6 ng/mL with clonidine loading.

**Figure 2:**
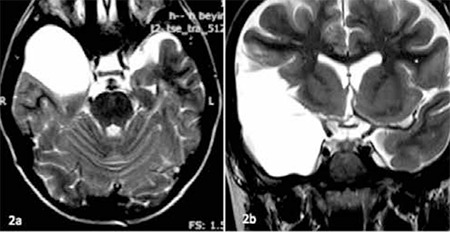
a,b) Axial/ Coronal MRI of patient 2 showing an extensive arachnoid cyst in the right middle cranial fossa, there was any compressive imaging findings (T2 weighted image)

Due to the history of a traffic accident and intracranial AC, the brain MRI was repeated and a extensive right middle fossa AC (MFAC) was detected with no compressive imaging findings (Figure 2a and 2b). We decided to treat the patient with recombinant human GH replacement therapy (0.025 mg/kg/d). His height increased by 9.2 cm in the last year, IGF-1 level was raised up to 274 ng/mL. There was no complication related to treatment in follow-up period. The control MRI scan 1 year after start of treatment showed no change in the cyst size.

## DISCUSSION

Intracranial ACs are usually asymptomatic and are identified incidentally during intracranial imaging. However, most patients with ACs (95%) are diagnosed in early childhood due to neurological symptoms and the remaining of them (5%) are diagnosed due to endocrine disorders (2,9).

Clinical symptoms show variability depending on the localization and size of the cyst. Therefore, endocrine disorders such as central precocious puberty (CPP), GH deficiency and amenorrhea are associated commonly with suprasellar ACs (8,10). Rarely, MFACs can be also cause of endocrine disorders. So far, only few such cases have been reported in the literature.

Mohn et al (8) reported follow-up of six patients with ACs. Two of them had a temporal AC, one had GH deficiency and another suffered from precocious puberty. Onal et al (11) identified endocrine disorders associated with MFAC in 3 of 13 pediatric patients. One of them had precocious puberty, while the others had GH deficiency.

It is still unknown how ACs cause endocrine disorders. This association can be possibly explained by the pressure-related compression or direct mass effect of the cyst on the hypothalamus-pituitary axis (12). MFACs, particularly large cyst extending to the suprasellar region, may also cause endocrine disorders with similar effect (13). However, further investigation is needed to confirm the pathological relationship between MFAC and endocrine disorders, particularly any finding of pituitary stalk compression or stalk deviation (8,11). Therefore, during determining the treatment options, physicians should consider that the real cause may be not the cyst itself but masked by the cyst. Surgical intervention is required when cyst causes either a mass effect or neural compression and if there is no other possible reason found for the patient’s symptoms (14). If endocrine disorders are not associated with the above-mentioned reasons and it is considered as a hypothalamic-pituitary dysfunction, then a hormonal evaluation and replacement therapy can be implemented (15). Our patients were diagnosed during the investigation of the etiologies of precocious puberty and short stature. One of them underwent surgery, because it was previously thought that the clinical findings were associated with mass-related compression effects of the cyst. After surgery, MRI follow-up showed a remarkable reduction of the cyst size. His puberty progression also regressed within one year. However, surgery was not considered as the first choice of treatment in the second patient, because, the pressure-related compression effect of the cyst on the hypothalamic-pituitary axis was not a likely cause. Moreover, there is an extremely low incidence of GH deficiency associated with MFAC. Hence, isolated GH deficiency is more likely diagnosed in this patient. Subsequently, we started treatment with recombinant human GH and no complication was observed during the treatment follow-up. However, the long-term effect of GH replacement therapy is also unclear for patients with ACs. We need more clinical experience before the effectiveness and safety of GH replacement therapy can be established (8,10,16).

In conclusion, coexistence of ACs with endocrine disorders should be kept in mind and endocrine outcomes should be evaluated accordingly. However, during determining the treatment options, physicians should consider that the real reason may be not the cyst itself but may be masked by the cyst. Therefore, each case should be individually assessed clinically and radiographically, appropriate treatment options should be determined and should be followed up.
